# Supplementary material for: Pyruvate Dehydrogenase Kinase 1 inhibition mediated oxidative phosphorylation enhancement in cartilage promotes osteoarthritis progression
Source: BMC Musculoskelet Disord. 2023 Jul 20;24:597. doi: 10.1186/s12891-023-06585-6 (PMC10357736; doi:10.1186/s12891-023-06585-6)
Supplement: Supplementary file 1 — Additional file 1. [file 12891_2023_6585_MOESM1_ESM.zip › MF.Gsea.1653627316242/GOMF_PHOSPHOLIPID_TRANSFER_ACTIVITY.html]

Details for gene set GOMF\_PHOSPHOLIPID\_TRANSFER\_ACTIVITY[GSEA]

|  || Dataset | 55457\_collapsed\_to\_symbols.cls.cls #OA\_versus\_normal.cls.cls #OA\_versus\_normal\_repos |
| Phenotype | cls.cls#OA\_versus\_normal\_repos |
| Upregulated in class | normal |
| GeneSet | GOMF\_PHOSPHOLIPID\_TRANSFER\_ACTIVITY |
| Enrichment Score (ES) | -0.50431913 |
| Normalized Enrichment Score (NES) | -1.473167 |
| Nominal p-value | 0.06896552 |
| FDR q-value | 0.20608272 |
| FWER p-Value | 1.0 |
Table: GSEA Results Summary

  

Fig 1: Enrichment plot: GOMF\_PHOSPHOLIPID\_TRANSFER\_ACTIVITY      
 Profile of the Running ES Score & Positions of GeneSet Members on the Rank Ordered List

  

| SYMBOL | TITLE | RANK IN GENE LIST | RANK METRIC SCORE | RUNNING ES | CORE ENRICHMENT || 1 | TRIAP1 | TP53 regulated inhibitor of apoptosis 1 [Source:HGNC Symbol;Acc:HGNC:26937] | 2163 | 0.342 | -0.1013 | No |
| 2 | ABCA3 | ATP binding cassette subfamily A member 3 [Source:HGNC Symbol;Acc:HGNC:33] | 3549 | 0.198 | -0.1703 | No |
| 3 | OSBPL2 | oxysterol binding protein like 2 [Source:HGNC Symbol;Acc:HGNC:15761] | 3906 | 0.164 | -0.1661 | No |
| 4 | CPTP | ceramide-1-phosphate transfer protein [Source:HGNC Symbol;Acc:HGNC:28116] | 4654 | 0.100 | -0.2047 | No |
| 5 | PLEKHA8P1 | pleckstrin homology domain containing A8 pseudogene 1 [Source:HGNC Symbol;Acc:HGNC:30222] | 5759 | 0.011 | -0.2882 | No |
| 6 | GLTP | glycolipid transfer protein [Source:HGNC Symbol;Acc:HGNC:24867] | 7113 | -0.089 | -0.3759 | No |
| 7 | MTTP | microsomal triglyceride transfer protein [Source:HGNC Symbol;Acc:HGNC:7467] | 7218 | -0.097 | -0.3651 | No |
| 8 | PLTP | phospholipid transfer protein [Source:HGNC Symbol;Acc:HGNC:9093] | 7412 | -0.112 | -0.3583 | No |
| 9 | PITPNA | phosphatidylinositol transfer protein alpha [Source:HGNC Symbol;Acc:HGNC:9001] | 8883 | -0.223 | -0.4290 | No |
| 10 | PRELID3B | PRELI domain containing 3B [Source:HGNC Symbol;Acc:HGNC:15892] | 9854 | -0.307 | -0.4447 | Yes |
| 11 | C2CD2L | C2CD2 like [Source:HGNC Symbol;Acc:HGNC:29000] | 10495 | -0.372 | -0.4220 | Yes |
| 12 | PITPNM1 | phosphatidylinositol transfer protein membrane associated 1 [Source:HGNC Symbol;Acc:HGNC:9003] | 10641 | -0.387 | -0.3580 | Yes |
| 13 | PITPNM3 | PITPNM family member 3 [Source:HGNC Symbol;Acc:HGNC:21043] | 10658 | -0.389 | -0.2837 | Yes |
| 14 | PRELID3A | PRELI domain containing 3A [Source:HGNC Symbol;Acc:HGNC:24639] | 10954 | -0.425 | -0.2240 | Yes |
| 15 | PITPNB | phosphatidylinositol transfer protein beta [Source:HGNC Symbol;Acc:HGNC:9002] | 11527 | -0.505 | -0.1702 | Yes |
| 16 | PITPNC1 | phosphatidylinositol transfer protein cytoplasmic 1 [Source:HGNC Symbol;Acc:HGNC:21045] | 12349 | -0.691 | -0.0996 | Yes |
| 17 | CERT1 | ceramide transporter 1 [Source:HGNC Symbol;Acc:HGNC:2205] | 12463 | -0.732 | 0.0339 | Yes |
Table: GSEA details [plain text format]

  

Fig 2: GOMF\_PHOSPHOLIPID\_TRANSFER\_ACTIVITY      
 Blue-Pink O' Gram in the Space of the Analyzed GeneSet

  

Fig 3: GOMF\_PHOSPHOLIPID\_TRANSFER\_ACTIVITY: Random ES distribution      
 Gene set null distribution of ES for **GOMF\_PHOSPHOLIPID\_TRANSFER\_ACTIVITY**
